# Embodied Cognition With and Without Mental Representations: The Case of Embodied Choices in Sports

**DOI:** 10.3389/fpsyg.2019.01825

**Published:** 2019-08-07

**Authors:** Markus Raab, Duarte Araújo

**Affiliations:** ^1^Department of Performance Psychology, Institute of Psychology, German Sport University Cologne, Cologne, Germany; ^2^Sport and Exercise Science Research Center, School of Applied Sciences, London South Bank University, London, United Kingdom; ^3^Department of Sport and Health, CIPER, Faculdade de Motricidade Humana, Universidade de Lisboa, Lisbon, Portugal

**Keywords:** embodied cognition, ecological dynamics, common coding, affordance, representation, self-organization

## Abstract

In this conceptual analysis contribution to the special issue on radical embodied cognition, we discuss how embodied cognition can exist with and without representations. We explore this concept through the lens of judgment and decision-making in sports (JDMS). Embodied cognition has featured in many investigations of human behavior, but no single approach has emerged. Indeed, the very definitions of the concepts “embodiment” and “cognition” lack consensus, and consequently the degree of “radicalism” is not universally defined, either. In this paper, we address JDMS not from a rigid theoretical perspective but from two embodied cognition approaches: one that assumes there is mediation between the athlete and the environment through mental representation, and another that assumes direct contact between the athlete and the environment and thus no need for mental representation. Importantly, our aim was not to arrive at a theoretical consensus or set up a competition between approaches but rather to provide a legitimate scientific discussion about how to explain empirical results in JDMS from contrasting perspectives within embodied cognition. For this, we first outline the definitions and constructs of embodied cognition in JDMS. Second, we detail the theory underlying the mental representation and direct contact approaches. Third, we comment on two published research papers on JDMS, one selected by each of us: (1) Correia et al. (2012) and (2) Pizzera (2012). Fourth, following the interpretation of the empirical findings of these papers, we present a discussion on the commonalities and divergences of these two perspectives and the consequences of using one or the other approach in the study of JDMS.

## Introduction

Thinking about embodiment is as old as psychology itself. One of the founders of psychology, William James, stated early in his psychological writings that.

the world experienced comes at all times with our body as its center, center of vision, center of action, center of interest. Where the body is is “here”; when the body acts is “now”; what the body touches is “this”; all other things are “there” and “then” and “that.” (James, 1890, p. 154).

We argue that when embodied choices in complex environments such as sports are considered, taking two recently discussed theoretical approaches into account can be useful. One of them assumes that there is mediation between a person and the environment through mental representation, and the other assumes direct contact between a person and the environment and thus no need for mental representation. Both approaches will be used to contrast interpretations of embodied cognition in sports. This paper is submitted as part of the special issue on radical embodied cognition, and thus, we first define and position these theoretical approaches in this discussion, then provide a detailed analysis of these divergent theoretical positions, and finally reexamine empirical evidence in two published articles from the perspective of the approach that was not followed in the original analysis.

Back in 1890, James was an early voice attempting to challenge what was then the accepted separation of the body and the mind, as put forth by Plato (428/427 to 348/347 B.C.) and, more explicitly, by Descartes (1596–1650). Today, if one searches for the term “embodiment” or “embodied cognition,” more than 10,000 papers from the last 50 years and 100 of reviews in a dozen disciplines can be retrieved from the Web of Science, a body of literature that is growing at a rate of at least three papers a day. This trend shows that the consideration of motor control, and thus the importance of the body and its movements, is now far from being the “Cinderella of psychology” (Rosenbaum, 2005) it once was.

Radical embodied cognition approaches assume that the functioning human body itself constitutes a cognitive process (Chemero, 2011; Jacob, 2016). According to Jacob (2016), there are at least two approaches. The “basal radical” approach principally denies the existence of representations and challenges computational approaches to cognition. We refer to this as taking a direct contact approach. The “constructive radical” approach describes bodily processes with regard to their functionality and as a component of cognition. In some cases, a constructive radical approach accepts that there can be a mediating role of mental representation (see Newen et al., 2018, for a range of positions). In sports, this discussion can be traced back to the motor-action controversy (Beek and Meijer, 1988), in which a rather representational approach based on motor program theory (Schmidt, 1988) and an ecological approach to movements (Reed, 1988; Warren, 1988) that excludes representations from its explanations were pitted against each other.

It should be noted that we, the authors, differ in regard to the radicality of our embodied cognition perspectives. We agree that the mental representation approach could just as well be defined as a moderate embodied cognition perspective, contrasting with the more radical direct contact approach. Below, we define and elaborate of the concept of representations. From the moderate perspective, cognitive processes are based on or are at least moderated by sensorimotor processes (Barsalou, 2016; Zona et al., 2018). The conceptual basis of this paper is that empirical findings indicating effects of sensorimotor processes on cognitive processes cannot be ignored (Goldinger et al., 2016) or explained as an “epiphenomenon” (Topolinski, 2010). Further, following the frequently cited review “Six views of embodied cognition” (Wilson, 2002), we agree on Wilson’s major assumption of cognitive processes being situated, dynamic, and functional and serving actions or being expressed by actions. The mental representation and direct contact approaches differ in whether they see a strict separation of the environment and the person. In the following, we argue that an embodied cognition perspective is needed to understand the dynamic interplay between people and the environment.

The present conceptual analysis focuses on the level of the individual (or group) performance domain (Rohrer, 2007) using mainly observed behavior of individual athletes in sports. Further, we define the representational and direct contact embodied cognition perspectives (see next section) such that an organism is a direct contact agent to the extent that its behavior is an immediate (not mediated by mental representations) function of its ongoing interplay with the environment by perception-action coupling and “a representational decision maker to the extent that its behavior is an immediate function of various higher-level states downstream from its perceptual states” (Schulz, 2018, p. 14).

## Representing the World and the Body or Contacting the World with the Body: Contrasts Between Embodied Cognition Perspectives

### Clarification of the Notions of Representation and Person-Environment System

In the present paper, we refer to embodied cognition as: “Cognition is embodied when it is deeply dependent upon features of the physical body of an agent, that is, when aspects of the agent’s body beyond the brain play a significant causal or physically constitutive role in cognitive processing” (Wilson and Foglia, 2017, p. 1). When considering judgment and decision-making as embodied cognitive processes, we refer to the concept of embodied choice: “The central statement of embodied choice is the existence of bidirectional influences between action and decisions. This implies that… the action dynamics and its constraints (e.g., current trajectory and kinematics) influence the decision-making process” (Lepora and Pezzulo, 2015, p. 1). We extend this definition by adding that also previously learned movements can influence current decisions (Raab, 2012).

About the notion of representation, it is important to differentiate the representational and nonrepresentational perspectives. We argue that representations within the research field of embodied cognition represent “neither states of the body per se nor states of the environment per se, but rather relations between body and goal” (Pacherie, 2018, p. 377). Naturally in science, there are multiple variations of such definitions as well as levels to describe them (Bickhard, 1998; Haselager et al., 2003). For the context of this paper on embodied cognition, we do not use a narrow concept of representations, considering them amodal or encompassing abstract symbols as often discussed in computational-representational theories of cognition (see Dempsey and Shani, 2015 for a discussion). We rather follow what is called an action-based approach: “An action-based approach to the problem of representation holds actions play a central role in shaping cognition” (Dempsey and Shani, 2015, p. 833). The content of such representations does facilitate prospective regulations of actions and can be discussed for instance as common codes (Prinz, 2013), or events (Hommel, 2015), instantiated at a neuronal level as a group of mirror neurons (Rizzolatti and Craighero, 2004).

The notion of person-environment system has implications for the concept of mental representation. Some approaches to psychology tend to be based, tacitly and explicitly, in a number of dualisms of which mind-body is the most common, thus the need for an “embodied cognition” manifesto. For ecological psychology (e.g., Richardson et al., 2008), these multiple dualisms are reflections of an overarching dualism: the view that organism (such as persons and other animals) and environment are logically distinct, separate systems (Järvilehto, 1998; Turvey and Shaw, 1999). The dualist view localizes cognitive processes in one’s mind and brain not in the surrounding. Consequently, a separation between person and environment originates explanations of cognitive activity centered at the organism. However, when organism is considered separate from environment, and the partial system (organism) represents the whole system (i.e., environment and organism: representations that give meaning to the stimuli from the environment and representations of how to control organism’s actions), there is a tendency to find explanation for behavior through variables (mental representations) that are beyond direct observation (Richardson et al., 2008). Contrary to this tendency, for the ecological approach (Gibson, 1979), the person and environment are mutual (one implies the other) and reciprocal (one could not exist without the other), in that the existence and influence of organism on environment and the existence and influence of environment on organism are both equivalent and complementary (Gibson, 1979; Richardson et al., 2008). More than just mutual and reciprocal, however, organism and environment are a combined whole (Turvey, 2009), such that the organism-in-its-environment (i.e., the organism-environment system) should be taken as the proper unit of analysis for studying behavior (Turvey and Shaw, 1999). Järvilehto (1998) suggests that, from this perspective behavior is a reorganization of the organism-environment system, not an interaction of organism and environment; and cognitive processes are different aspects of the organization and dynamics of the organism-environment system, not local processes of the organism. This is why from an ecological approach, there is no need for a part of the system (the organism) to represent the other part, or to represent parts of itself (the body), or representing the interactions between both parts (for example, see Vicente and Wang, 1998, or Turvey and Shaw, 1979, for memory without mental representations; see Turvey and Carello, 2012, for intelligence without mental representations).

### A Moderate Embodied Choice Perspective: Embodied Cognition With Mental Representation

In a recent review of different embodiment theories (Gentsch et al., 2016), one representational approach identified was the common coding principle (Prinz, 2013), a prototypical account of embodied cognition with mental representations (see Gentsch et al., 2016 for other representational accounts). It deviates from traditional representational accounts who argue for amodal and independent representations of cognition, action, and perception (Block and Fodor, 1972, for an overview, see Newen et al., 2018). Instead, perception and action are linked by a “common code,” meaning that perceptions can be transformed in the mind directly into actions and actions can influence perceptions (Prinz, 2013). The common coding principle is based on James’s hypothesis about the anticipation of action goals: That is, anticipated consequences (those to be perceived in the future) guide people’s actions (see Mechsner et al., 2001, for an empirical demonstration).

In contrast to common coding, other radical embodied cognition perspectives are anti-representational (Wilson, 2002; Chemero, 2011), but this rejection of representations when explaining decision-making has recently been criticized (e.g., Schulz, 2018). Consequently, other theories have been developed that integrate embodied cognition with a representational account of decision-making (e.g., Schulz, 2018). However, in a recent discussion, representational accounts of decision-making have been associated with costs of representations when deciding. If representations are costly arguments presented in detail in Schulz (2018) doubted that evolution has favored representations for decision-making. The typical argument that representational decision-making is costly is as follows (e.g., Schulz, 2018): Representational decision-making is costly, slow, and needs attention when humans decide. Given that nonrepresentational (i.e., direct contact) decision-making avoids such costs, it has been proposed that evolution favors it. Schulz (2018) refuted this argument by showing that representational decision-making can also be fast and frugal (Gigerenzer and Gaissmaier, 2011), allowing for adaptive behavior as well as neural efficiency and thus evolution favors both representation and nonrepresentational decision-making. One reason why representational decision making can be beneficial is that it allows for more flexibility in choices (Schulz, 2018). A second argument is that fast and frugal heuristics are quick and accurate (Gigerenzer et al., 1999). In short, “the fact that decision rules need to be stored does not affect the ease with which organisms can adjust to changed environments, and it does allow for faster and more frugal decision-making” (Schulz, 2018, p. 183). Thus, representational decision-making is not as slow as often assumed (see below, with the simple heuristic approach, how decisions can be fast and frugal). Finally, Schulz (2018) showed that much decision making is representational to allow for adaptive behavior in the many domains in which choices take place.

Common coding and direct contact alternatives can be compared by looking at the types of tasks in which representations might be needed. Representational decision-making might be more useful for decisions that are deliberate and protracted (e.g., an athlete’s decision to retire, made over the course of a month) than for short-term decisions (e.g., a playmaker’s allocation decision made in a split-second during control of the ball). This distinction is based on the work of Schütz-Bosbach and Prinz (2007). They separated offline effects from online effects in embodied cognition. Offline effects refer to the self-stored experiences of movements that influence a person’s own current decisions in a task even when the person is not moving. Online effects refer to the influence of current information from one’s moving body on one’s judgment. For example, a soccer coach observing the players on the pitch uses only her offline experienced movements to judge whether a pass or a shot is a good decision, but a player on the field additionally also uses his current (online) movement to decide to pass or shoot. Coaches’ and players’ behavior in principle can be explained by both representational and nonrepresentational decision-making. As argued above representational and nonrepresentational decision-making processes seem to have been equally favored by evolution (Schulz, 2018) and can both be used adaptively, depending on one’s current behavioral needs (see Raab, 2017, for modeling such dynamic and probabilistic behavior).

Representational decision-making is often assumed to rely on mental representations that are determined by content-specific models and perspectives (Gentsch et al., 2016). According to the common coding principle, perception and action share a “common code” and thus are represented together. On the neural level, this common code is reflected in a group of neurons, so called mirror neurons, that are active both when movements are observed and when movements are performed (Prinz, 2012; Barsalou, 2016). Formally, this can be denoted as “higher-level state S in organism O has content C if S has the appropriate structure to allow O to detect the world’s being in state C” (Schulz, 2018, p. 16).

How can these mental representations be used in decision-making in sports? Raab (2012, 2017, in press) has tested the simple heuristic approach and extended it to judgment and decision making in sports (JDMS). The simple heuristic approach is based on indirect perception: People perceive cues in the environment that they choose to use according to the cues’ validity, that is, how often a particular piece of information (cue) was helpful in good choices before (see Gigerenzer, et al., 1999, for formal descriptions, behavioral tests, and modeling approaches). Simple heuristics have three building blocks: a search rule, a stop rule, and a decision rule. Those building blocks are thus a demonstration of a representational approach to embodied cognition.

How does one apply simple heuristics to sport decisions? One approach has been formalized (see Raab and Johnson, 2004) and illustrated with a basketball example: Comparing two (or more) options (e.g., allocating the ball to different team members) with multiple cues (e.g., distance to the basket, distance to the next defensive player), the decision maker (i.e., playmaker) should start with the cue with the highest validity, for example, pass to the teammate who is closer to the basket. If two teammates have the same cue value, that is, both players are perceived about equally distant from the basket, move to the next cue, such as the distance between the teammates and the closest opposing defense player, and continue until one cue has a positive cue value, for example, one teammate is less defended, and then play to that teammate. This logic has been applied to different sport situations (Raab, 2012, in press). The problem of the costs of representational decision-making is partly solved in the simple heuristics approach because rules are fast and frugal but still mostly accurate (see Gigerenzer et al., 1999). The main argument for why heuristics are efficient cognitive processes has been empirically validated, for instance, in the less-is-more effect (Gigerenzer and Brighton, 2009; for further evidence, see Schulz, 2018, p. 171). The less-is-more effect indicates that the expected proportion of correct inferences is higher when less information is used to decide. A good example of the less-is-more effect is the recognition heuristic, which has also been applied to sports, for instance, predicting the winners of women’s and men’s tennis matches in the Wimbledon tournament (for an overview see Bennis and Pachur, 2006). Using the recognition heuristic, one would predict for each competing pair that the recognized player will win. Studies applying such a test of recognition to predict a competition’s outcome have so far usually compared the predicted against the actual outcomes of a game. They have shown that the recognition heuristic can describe betting behavior quite well (e.g., Serwe and Frings, 2006; Scheibehenne and Bröder, 2007). Furthermore, the accuracy of predictions based on recognition are equal to, or even better than, experts’ seedings (an equation to calculate rankings of all players using multiple and weighted parameters) by the tennis associations, which are based on much more complex algorithms and a greater amount of information (Scheibehenne and Bröder, 2007).

We now illustrate this point by discussing selected articles with embodied cognition in JDMS. The rationale for selecting prototypical empirical illustrations for the conceptual analysis is to prepare a comparison of the mental representation and direct contact perspectives of embodied cognition. If the bidirectionality between perception and action holds (common coding principle), then training that activates the motor system (blindfolded to avoid visual perceptual input) should result in better visual perception. Casile and Giese (2006) asked participants to learn awkward walking by setting the arm movements to a phase of 270° to the gait pattern which is very far of the normal gait behavior. After participants had learned this pattern, they were shown point-light displays of these movements and movements in which the degree of arm movement, and gait pattern was not 270° and were asked to identify movements (e.g., those that belonged to the class of 270° phase) or to discriminate stimuli (e.g., two movements are the same or different). Interestingly, participants from the motor-learning condition were better at identifying and discriminating movements without any visual-learning input. Good motor learners were better than poor motor learners in the perceptual identification and discrimination tasks, indicating that the quality of the learned movements influenced the perceptual judgments. We argue that these findings demonstrate, from an embodied cognition perspective, how the motor learning without visual input can influence visual task performance.

The empirical benefits of motor training in the above study for perceptual judgments can be interpreted from a common coding perspective as follows: A person seeing point-light displays of movements will predict the action effects of those movements. If the person has experience with those movements, the solely perception of movements will activate the corresponding action *via* the common coding. The additional activation of the motor experiences allows that person to perform well in the identification and discrimination tasks (for similar effects and explanations that refer to internal models from our laboratory, see Kennel et al., 2014a,b; for other laboratories, see Tucker and Ellis, 1998; Calvo-Merino et al., 2006).

Is empirical evidence from Casile and Giese (2006) enough to say that the motor system affects the visual perception of movements and thus is the action-execution part of the decision-making process? This question is presented because the learning experience in the Casile and Giese’s study is a typical offline effect of embodied cognition, as described above. The learning was prior to the perceptual tasks in which participants were not moving. Whether action execution is part of the decision-making process, when online movement choices are conducted, was investigated by Aczel et al. (2018) in arm movements toward two targets. The authors differentiated whether the movement to one of the targets was influenced by the ongoing movement toward the target or by the movement costs associated with changing the movement direction to select a different option. In two experiments, they showed that energetic movement costs influenced target choice but not the ongoing direction of the movement. The authors interpreted these experimental results as evidence against an embodied-choice hypothesis. We assume that the experimental evidence does not violate embodied-choice principles as the energetic costs of changing movement trajectories between two arm movements are small. However, in other real situations, such as climbing, for example, action execution would most likely be part of the decision-making process because the climber’s survival could depend on it. Another line of evidence is based on neuroscience showing that the motor system is active during deciding between two targets (Cos et al., 2011). In sum, we argued that a representational approach of embodied cognition could be used to interpret empirical findings of sport choices which we call an embodied choice perspective (Raab, 2012).

### Ecological Dynamics of Decision-Making: Embodied Cognition With Direct Contact

As recently mentioned by Baggs and Chemero (2018), basal radical embodied cognitive science has two approaches: the ecological approach and the enactivist approach. Both approaches reject representations as a needed construct to explain cognition. However, the ecological approach starts from the organization of the environmental properties (action possibilities) to explain how they constrain behavior for individual actors. The enactivist approach tends to start from the individual, characterizing the individual’s exploratory, self-regulating behavior. Here, we present a particular ecological framework that combines dynamical systems with Gibsonian ecological psychology: the ecological dynamics framework (Araújo et al., 2006, 2017). This approach stresses the primacy of individual-environment relations in understanding cognitive processes. The link between a performer and his or her environment is the proposed starting point for understanding how performers move about, select routes, decide with whom to cooperate, and compete with adversaries in the actual competition environment (see Correia et al., 2013, for a review of studies exemplifying how). In the representational view, the behavioral expression of those decisions is not at the heart of cognition because behavior is assumed to be an implementation of a mental representation (see Adams, 1987 for a review).

For ecological dynamics, the regulation of behavior as an expression of a cognitive process (Gibson, 1979) should not be attributed to one part of the individual-environment system (i.e., the performer) but to this entire system. The selection of actions is embodied (i.e., it is shaped by the skills and characteristics of the body) and embedded (i.e., the performer-environment system as the unit of explanation) in the performance context; that is, it is not based on inferences or other mental processes but on the interplay between whole-body action and perception in a sport performance environment (Araújo et al., 2017). Cognition is embodied (in a body) and embedded (in a context) so that detecting event information related to spatiotemporal characteristics specifies body forces and torques required in goal-directed action (Richardson et al., 2008). This understanding of cognitive processes embraces the notion that major influences shaping individuals’ behavior are from the social and physical environment, as well as from their own action-perception skills (Araújo et al., 2017).

In ecological dynamics, decision-making behavior is defined as transitions in a course of action, where cognitive processes are necessarily constrained by the evolving environment-individual system (Araújo et al., 2006; see below). The current state of this system is the result of a history of interactions that constrain the immediate action. In this way, current performance is shaped by memory and past experience (memory need not be based on representations; Gibson, 1994; Reed, 1996). An individual’s behavioral history as well as the skills and characteristics of the body channel action to a landscape of possibilities for behavior (affordances) offered by a particular environment. The field of affordances reflects the multiple possibilities for action that stand out as relevant for each individual in a particular situation because of his or her specific training, skills, and experience in related tasks (Rietveld et al., 2018). This means that the affordance landscape is constrained not only by the past (e.g., what is in memory) but also by the future, that is, by task goals, actions’ path dependency toward which the current action may be directed (not necessarily expectations or beliefs about the future). Behavior is an expression of skill, and at the same time, it is an expression of how the environment draws the individuals into it and solicits actions (Gibson, 1994; Reed, 1996).

Measures of action, therefore, are a direct expression of cognition, contrasting with indirect expressions of behavior (and cognition) such as neurophysiological or verbal correlates (Araújo et al., 2017). Neurophysiological and verbal correlates are not measures of cognition. They are nervous systems activations or reported words from which researches make inferences about cognitive processes. In turn, action is directly measurable, and if action is an expression of cognitive processes, then cognitive activities can be directly measured. Skillful behavior is constrained by its past (path dependency) and its future (affordances), resulting in action being an ecologically flexible process (self-organized, emergent) to satisfy impinging constraints. When a system establishes a state due to the dynamic interactions among elements within the individual-environment system (no element guiding the organization), the state is self-organized. Self-organization, as the term suggests, is not caused by external (e.g., coaches’ instructions) or internal (e.g., the mind) processes, but it is instead generated by interacting constraints within the individual-environment system (not directed by one single constraint). Actions (e.g., a lateral pass in soccer) that emerge (when a pass line suddenly appears) are different from the components that make up the system (e.g., the pitch, the ball, teammates, the legs of the performer) and cannot be predicted solely from the characteristics of those components. Consequently, many sorts of solutions to achieve a sport task goal can emerge given the many different ways its elements can interact under the same constraints. But instead of being a random process, or in the other extreme, a process that is internally programmed (determined) in advance, performers are perceptively attuned to affordances (by detecting information) that guide self-organizing action toward achieving a task goal (Davids and Araújo, 2010).

A major challenge is to understand the ability of individuals to perceive the surrounding layout of the competitive environment on the scale of their own body and action capabilities (Fajen et al., 2009). As performers move with respect to their surroundings, opportunities for action persist, emerge, and dissolve, even if the surrounding environment remains stable. Subtle changes of action can give rise to multiple and marked variations in opportunities for subsequent actions. The dynamic process implied in the perception of affordances provides the basis by which performers can control their behavior prospectively (Montagne, 2005). However, cognition has traditionally been defined as the information processing that produces mental representations, even though there are no direct experimental observations of internal representations (i.e., representations cannot be directly measured). Probably, the definition of cognition proposed by Stepp et al. (2011) best captures the embodied-embedded nature of the ecological dynamics perspective: “Cognition is the ongoing, active maintenance of a robust organism-environment system, achieved by closely coordinated perception and action” (p. 432). This understanding of cognition emphasizes its nature as an activity and its close relationship with perception and action. From this ecological perspective, characteristic cognitive capabilities accommodate the physical principles of dynamic systems (involving time evolution of observable quantities according to, for example, thermodynamic principles). Dynamical systems can offer tools (e.g., nonlinear physics and differential equations) to understand cognitive processes (see Araújo et al., 2014, for an example of mathematical modeling of dynamic decision making in rugby using differential equations, and see Araújo et al., 2019, for a review of studies in sport performance).

An important nuance is that affordances are only accessible to individuals with the necessary skills to act on them. This is why the characteristics of a performer, such as skill level, are of paramount importance. Rietveld et al. (2018) defined “skilled intentionality” as the performer’s selective openness and responsiveness to a field of affordances. During the act of perceiving, the hands, legs, ears, or eyes of a performer can explore the available information in an environment, searching the surrounding, structured energy patterns. These structured energy patterns (i.e., information as it is ecologically defined), such as the light reflected from the soccer ball, are an environmental resource to be exploited by active players (Reed, 1993). From this viewpoint, the process of perceptual attunement brings a readiness to affordances that without skill would not be accessible, since it is skill that opens possibilities for action to an individual (Rietveld et al., 2018). This implies that while affordances may exist in a performance or practice context, athletes’ skills facilitate their use of specific affordances (which invite actions, Withagen et al., 2012). Importantly, successive actions are modulated by the individual exerting her or his agency by intentionally driving, the performer-environment system dynamics at appropriate points to yield a trajectory (Withagen et al., 2017). In turn, these local dynamic interactions are coupled to larger scale dynamics, guiding the formation of the behavioral trajectory over longer time scales.

Reciprocally, the longer term dynamics could influence the short-term interactions (and thus highlight specific affordances), for example, by altering environmental conditions. Because a behavioral trajectory is assembled anew on each occasion, the action sequence is historically contingent and variable, allowing for the flexibility observed in ordinary action sequences. From this viewpoint, decision-making emerges as athletes search in a field of affordances to arrive at a stable, functional solution, emphasizing the performer-environment reciprocity (e.g., Araújo et al., 2014).

## Contrasting Interpretations of Results of Embodied Cognition

In the following, we evaluate two JDMS papers that were chosen to serve as prototypical examples of the perspectives on embodied cognition and their contrasting interpretations of effects discussed in this article. This allows us to illustrate our argument that the interpretation of empirical results can be based on either concept. Each paper is an example of decision-making research in sports using one of the embodied cognition perspectives. Both papers demonstrate how research questions are formulated in the JDMS domain from an embodied perspective, provide phenomena that need explanation, and present two established paradigms that are used for research in JDMS. More specifically, the paper by Correia et al. (2012) was chosen because it tests a nonrepresentational hypothesis by means of assessing the dynamics and organization of action patterns for explaining choices. The paper by Pizzera (2012) was chosen as it provides a theoretical background of a representational embodied cognition approach using discrete choices and long-term motor experiences as the source of the judgmental process. Choosing these papers allows us to contrast the approaches and leads to a discussion of their commonalities and divergences in the “General Discussion and Conclusions.”

### Comments on Correia et al. (2012)

The study by Correia et al. (2012) showcases the dynamic systems approach within JDMS from a direct contact perspective using skilled young rugby players. In this study, the distance between two defenders in rugby influenced the movement parameters of the attacker and the probability of the outcome being to tackle (defender wins) or to try (attacker wins). The authors’ explanation was that movements are used flexibly and that movements adapt to changes in the environment such as the defenders’ behavior. The authors further argued that the defenders’ movement displacement trajectories express different “preferred relational states” in this attacker-defender system and that the attacker reacts to the defenders’ movements. Further, they argued that decision-making behaviors (e.g., the decision where and when to run) emerged as a function of changes in attackers’ spatial location during the performance.

From an embodied choice account of embodied cognition (Raab, 2017), the environmental constraints (distance of defenders) would be used by the attacker to predict the action consequences of his own movements, which would be updated dynamically. A choice to move in one of the movement directions to pass a defender can be modeled as an embodied choice as follows: The attacker uses predictions to prepare his movements. This means in terms of common coding, the anticipated response consequences that are based on his own sensorimotor representations. The hypothesis of the study would remain the same: Experts will be good at predicting the outcome of specific choices. The interpretation of the results, however, would change: The explanation of what movement will be produced and how would be based on the interaction of the sensorimotor and the cognitive system and would rely on a representation in a common code between perception and action.

From the simple heuristic perspective, the perception of the distance to defenders is defined as a valid cue for making the choice. The use of the cue changes dynamically depending on the speed and the movement trajectories of the attacker. If the cue “largest distance to the next defender” is the most valid cue, the athlete would choose the direction that would increase the distance to the next defender. Another interpretation is that the distance between an attacker and defenders is used by rugby players to predict players’ actions and internally model them (see Raab and Johnson, 2004 for an experiment and a computational model in basketball).

### Comments on Pizzera (2012)

Pizzera (2012) studied regional gymnastic judges and the quality of their ratings of gymnastic performances. The task of these judges was to perceive and evaluate gymnasts’ performances according to predefined scoring rules. In her introduction, Pizzera highlighted that research has shown that perceptual judgments can be constrained by judges’ position in respect to the gymnast and that the ability to perceive the information sources is another key aspect. This attunement to sources of information seems to be developed by exploring the task through different means (i.e., by performing it and by observing others perform it). Pizzera’s study aimed to clarify if judges who had performed the judged tasks and/or had experience observing others perform such tasks would achieve higher quality in judging gymnasts’ performance in such tasks than someone without this motor and observational experience. The assessment of such experience was based on a questionnaire that asked, for instance, how many years they had performed gymnastics, how many times they watched gymnastics per week, and if they could perform a specific task on the balance beam.

After judges learned how to use an online video test at their personal convenience, they rated gymnasts performing a specific balance beam task. The quality of the judgment was compared between judges who could perform the task themselves and those who could not. Pizzera found that adding general motor, visual, and judging experience did not improve the prediction of judgment quality. But judges benefited from their own motor experience on the specific balance beam task, increasing the quality of their judgment. Specifically, Pizzera found that judges who practiced the specific task they were asked to judge focused “on aspects that allowed them better perceptual sensitivity” (p. 606), and she suggested that studies focusing on eye-movement strategies may clarify the mechanism underlying this advantage. Pizzera concluded that this development of judgment expertise was achieved through structured and effortful adaptation produced by training.

From an ecological dynamics perspective, we could not agree more with this explanation of the findings of this study. Indeed, to understand how a specific performance is achieved, one has to understand how the performer interacts with the task, and how the history of such interactions (experiences produced by training) perceptually attunes the performer to the relevant sources of task information that allow the performer to attain task goals. This is an explanation fully aligned with ecological dynamics, in that it is based on embeddedness and embodiment of the motor experience, in this case acting to perceive, attuning the judges to the relevant task information sources.

What is somewhat unclear, in the sense that it does not follow from the previous line of argumentation, is the second part of Pizzera’s discussion. She argued that the results from the study suggest that judges “use their personal experiences as information” (see abstract, p. 603) to improve the quality of their perceptual judgments about gymnasts’ performance in the studied task.

Why would a judge’s perceptual sensitivity to the environmental information presented by a gymnast performing a specific task need to be combined with another, apparently unrelated, source of information, namely, personal experience from the past? A more parsimonious explanation would be to hypothesize that performance judgment is based on perceiving what is relevant from the environment directly, rather than perceiving this information and then, by means of some obscure process (that cannot be directly measured), combining it with some consulted information from the past, with the goal of making the externally detected information more accurate—exactly the same information that had been detected directly since the beginning. Then Pizzera continued to emphasize this less parsimonious explanation: “It would be interesting to investigate whether judges with and without motor experience differ in how they internally represent the performed routine or memorize the skills” (p. 607).

There may be some difficulties following through on this suggestion. The first is how to scientifically capture how judges internally represent the performed routine and how to detect differences in mental representations. Would it be based on indirect measures of what mental representations might be, such as brain activation areas or verbalizations about such mental processes? (see Araújo et al., 2017, for a discussion). Then one would need to justify how such mental representations are related to the task that the judges are performing (how the internal represents the external), how using such internal information benefits the perception of the gymnast’s performance, and finally how such internal and external sources of information are combined. Beyond the demonstration of such processes, there is another difficulty, which is to justify why such a detour (i.e., consulting internal information and then combining internal and external information) is necessary, if what seems to explain expertise is precisely the perceptual attunement to the relevant sources of (external) information (the gymnasts performing the task). This capacity to make high-quality perceptual judgments was achieved by the judges interacting with the environment over time, by means of a structured and effortful adaptation (attunement) to the task environment (motor and observational training), as Pizzera indicated. So, why search for mental representations if expertise in perceptual judgment is based on the discrimination of and sensitivity to environmental sources of information, developed through the particular bodily interactions with the performance environment? In short, a very straightforward explanation can be provided by an ecological dynamics approach to decision-making, namely, that judges with experience in perceiving and acting (i.e., performing) a task are perceptually attuned to the relevant constraints for performing that task.

## General Discussion and Conclusions

It should now be obvious that there are some commonalities and key divergences in the two embodied cognition approaches presented here. Starting with the theoretical commonalities, there is the body as the starting point for understanding cognition. For both approaches, the body shapes the knowledge of the world, where perception and action, that is, the means by which organisms contact the world, are intimately linked. The mental representation and direct contact perspectives of embodied cognition share the understanding that choices are not idealistically rational, and humans do not search for optimization or follow optimal rationality norms. Rational choices as advocated in some areas of psychology ignore bodily information for decision-making and thus are too mechanistic to be useful for JDMS. We envision a perspective here that highlights embodiment as a key to understanding JDMS, a perspective that defines success not in terms of optimality but in terms of adaptiveness in a current situation.

It is on how the body contributes to this ecological success that the two approaches diverge. For the embodied choice perspective, there is a place in the body (the brain) where perceptual representations match with action representations (i.e., common coding principle). But for the ecological dynamics (i.e., direct contact) perspective, there is no need for such representations because perception (and action) is direct (not mediated by representations): The patterns of ambient energy (i.e., information), such as light reflected from objects, directly inform what is there for organisms capable of detecting such energy patterns (e.g., humans do not have perceptual systems that detect infrared light). And therefore, action enables perception, and perception enables action.

Theoretically, both perspectives should be able to explain how long-term experiences with specific movements help to advance peoples’ decision-making in sport and more specifically how referees, athletes, and coaches decide. Correia et al.’s (2012) study was originally presented from an ecological dynamics perspective. Here, we interpreted the results from an embodied choice perspective. Pizzera (2012) interpreted her findings from a common coding, representational perspective as part of a research program in her dissertation. The alternative interpretation argued from a nonrepresentational perspective. Both studies were used for the present conceptual analysis to simply illustrate the starting point for theorizing, rather than demonstrate a falsification approach that can be accepted or rejected by an empirical test. The conclusion of this conceptual analysis is that mental representation and direct contact theoretical accounts of embodied cognition can coexist when explaining JDMS, to further refine the different theoretical views on embodied choice phenomena that are far from being understood.

From a methodological point of view, the two approaches converge in the search for representative experimental task designs (Brunswik, 1956), in the sense that the tasks represent the circumstances about which the findings are aimed to generalize. However, for the embodied choice perspective, the measurements are mainly discrete (e.g., the score of the judge’s quality in each trial), whereas for ecological dynamics, the measurements are mainly dynamic (e.g., the time series of each trial’s trajectory of an attacker when facing the defenders near the try line). This methodological difference also highlights a theoretical difference: that decisions are not all-or-nothing snapshots but continuous adjustments and transitions of a performer within his or her environment (Correia et al., 2013). On the other hand, current models in JDMS are often probabilistic and dynamic (e.g., see Raab and Johnson, 2004, for a dynamic representational approach in JDMS).

Another line of divergence is the use of the representative design concept itself. From the perspective of the simple heuristic approach to embodied cognition, representative design (see Brunswik, 1956) refers to the tasks in an experiment needing to be randomly selected (as participants are) from the general distribution and class of tasks in the environment. From the ecological dynamics perspective, representative design means that the actions and environmental constraints need to be representative of the person-environment interactions in the real world (Pinder et al., 2011). We recommend providing a more detailed description of task selection for designing studies (see Johnson and Raab, 2003) or including environmental constraints (Chow et al., 2011) such as time pressure within a task (Musculus et al., 2018).

From a practical point of view, the authors agree that, for instance, for a coach, the implementation of these theoretical perspectives in practice may be overall similar. Consider some practical implications of Correia et al. (2012), p. 249: (1) “Participant behaviors are flexible and adapted in a goal-directed manner to current task constraints” and (2) “simple practice task constraint manipulations, such as varying number of players involved, distances between players (e.g., defender-defender initial conditions) and field dimensions, powerfully influence emergent decisions and actions of performers (attackers and defenders) in team games.” What does taking a mental representation or a direct contact perspective mean for selecting tasks in experiments and in training? Both perspectives would potentially agree that training needs to dynamically produce actions under constraints. This is in contrast to modular approaches to training, such as implicit perceptual training (Jackson and Farrow, 2005), cognitive training, such as in steps-of-decision-making training (Vickers, 2007), and repetitions of movement, such as out-of-water swimming movements. One potential distinction would be that for the embodied choice approach, a relevant part of an exercise would be to ask the performer to verbally generate options for the next action in a particular task, whereas for the ecological dynamics approach, the focus would be on acting upon perceived affordances; that is, instead of highlighting the generation of action options, the performer should perceive task affordances and act upon them. For example, Seifert et al. (2017) demonstrated that previewing a climbing route allowed climbers to become perceptually attuned to affordances. Once acted upon, they applied adjustments and revealed new information that, in turn, indicated further adjustments and so on toward goal achievement. Implementing these theoretical perspectives in experiments and practice sessions in sports could lead to a better understanding of individual differences in performance through the systematic use of multiple task variants and multiple tests (training measures), somewhat in line with recent methodological recommendations in the study of cognition (e.g., Boogert et al., 2018).

How can JDMS be improved by applying embodied cognition with and without representation? We illustrate this process with talent selection and development. Talent selection refers to sport systems in which youth are selected to become professional. In German soccer, this process involves about 2 million boys, of whom only 600 are selected by the youth academies of soccer clubs, and only about 30 receive a contract with a professional club thereafter. Talent development refers to the training, education, competitive team assignment, and transfer policies affecting these young players over time until they become professionals (de Oliveira et al., 2014).

From the mental representation perspective of JDMS, concrete applications need to refer to a specific framework. For instance, from the simple heuristics referred to above we would select and train talent on learning and selecting between different heuristics (de Oliveira et al., 2014). This talent strategy would not test cognitive and motor processes in isolation but rather would test sport- and context-specific heuristic use, often with separate measures of perception and attention (search and stop rules in heuristics), “what” decisions (which movement to perform), “how” decisions (how to perform a movement), and motor performance (decision and execution rules in heuristics). A talent selection camp in which decisions to select or not to select a talent would be based on a list of heuristics. Knowing for each relevant heuristic how well talent is able to use it at a specific age makes it possible to use the heuristic for talent selection (Musculus et al., 2018). For talent development, this simple heuristic perspective would not isolate cognitive and motor training but would integrate them (Jackson and Farrow, 2005).

The direct contact approach offers the principles for designing effective practice tasks and learning activities to develop talent and prepare them for the demands of competitive performance (Chow et al., 2011). In ecological dynamics, the development of judgment and decision-making in talented athletes is, in part, the result of their responsiveness to the design, types, and modes of activities experienced during practice and play (Araújo et al., 2004; Davids et al., 2017). Such activities offer the athletes the possibility to learn how to be adaptive in detecting information and realizing affordances in different performance environments. Gaining expertise in this important part of athletic development involves *selectivity* since not all affordances are “for good” for all performers (supporting goal achievement) and some can lead to problems such as negative outcomes, injuries, or poor health (Araújo et al., 2017). This connotation of affordances has some important implications for developing expertise and talent in sports. Competitive sports performance environments provide manifold action possibilities, which are uniquely relative to an individual (Gibson, 1979), requiring high levels of specific experience, development, skills, and intentionality to utilize such affordances (Rietveld et al., 2018). For example, opportunities for action in a performance environment that can be perceived and realized by a professional athlete will differ from those used by a recreational athlete (Chow et al., 2011; Davids et al., 2017). These ideas imply that task constraints, in an affordance landscape (Rietveld et al., 2018), can be designed to *invite* specific actions from different athletes, depending on personal constraints, such as skill levels. Therefore, creativity in practice task design is warranted in presenting affordance landscapes in sports that are dependent on the athletes’ expertise levels.

In conclusion, this conceptual analysis argues for a pluralistic account of the variety of processes implied in embodied cognition in sports. There is no single theory that can explain such complex processes and we follow what has been studied in science in general, that it may depend on the starting point of your theoretical account what and how researchers think representations are important within embodied cognition (see Hesslow, 2002; Svensson and Ziemke, 2005). The work presented may offer contexts and programs for further investigation, which can either contrast with or refine hypotheses from researchers accepting or rejecting mental representations. Despite our theoretical preferences, a hidden goal that brought us together was to focus the discussion on the explanation of the phenomena, highlighting that there are very different frameworks in place and that the discussion can go beyond computational power or sample size. The simple reason for this: JDMS is a fascinating domain to search for embodied cognition explanations.

## Author Contributions

The authors equally contributed to the structure, theoretical position, and writing of the manuscript.

### Conflict of Interest Statement

The authors declare that the research was conducted in the absence of any commercial or financial relationships that could be construed as a potential conflict of interest.
